# Prolactin and oxytocin: potential targets for migraine treatment

**DOI:** 10.1186/s10194-023-01557-6

**Published:** 2023-03-27

**Authors:** Anna K. Szewczyk, Samiye Ulutas, Tülin Aktürk, Linda Al-Hassany, Corinna Börner, Federica Cernigliaro, Michalis Kodounis, Salvatore Lo Cascio, David Mikolajek, Dilara Onan, Chiara Ragaglini, Susanna Ratti, Eduardo Rivera-Mancilla, Sofia Tsanoula, Rafael Villino, Karl Messlinger, Antoinette Maassen Van Den Brink, Tessa de Vries, on behalf of the European Headache Federation School of Advanced Studies (EHF-SAS)

**Affiliations:** 1 Doctoral School, Medical University of Lublin, Lublin, Poland; 2 Department of Neurology, Medical University of Lublin, Lublin, Poland; 3 Department of Neurology, Kartal Dr. Lutfi Kirdar Research and Training Hospital, Istanbul, Turkey; 4 Division of Vascular Medicine and Pharmacology, Department of Internal Medicine, Erasmus University Medical Center, Rotterdam, The Netherlands; 5 Department of Pediatrics – Dr. von Hauner Children’s Hospital, LMU Hospital, Division of Pediatric Neurology and Developmental Medicine, Ludwig-Maximilians Universität München, Lindwurmstr. 4, 80337 Munich, Germany; 6 LMU Center for Children with Medical Complexity – iSPZ Hauner, Ludwig-Maximilians-Universität München, Lindwurmstr. 4, 80337 Munich, Germany; 7 Department of Diagnostic and Interventional Neuroradiology, School of Medicine, Klinikum rechts der Isar, Technical University of Munich, Ismaninger Str. 22, 81675 Munich, Germany; 8 TUM-Neuroimaging Center, Klinikum rechts der Isar, Technical University of Munich, Munich, Germany; 9 Child Neuropsychiatry Unit Department, Pro.M.I.S.E. ”G D’Alessandro, University of Palermo, 90133 Palermo, Italy; 10 First Department of Neurology, Eginition Hospital, National and Kapodistrian University of Athens, Athens, Greece; 11 Department of Neurology, City Hospital Ostrava, Ostrava, Czech Republic; 12 Spine Health Unit, Faculty of Physical Therapy and Rehabilitation, Hacettepe University, Ankara, Turkey; 13 Department of Clinical and Molecular Medicine, Sapienza University, Rome, Italy; 14 Neuroscience Section, Department of Applied Clinical Sciences and Biotechnology, University of L’Aquila, 67100 L’Aquila, Italy; 15 Department of Neurology, 401 Military Hospital of Athens, Athens, Greece; 16 Department of Neurology, Clínica Universidad de Navarra, Pamplona, Spain; 17 Institute of Physiology and Pathophysiology, Friedrich-Alexander-Universität Erlangen-Nürnberg, Erlangen, Germany

**Keywords:** Prolactin, Oxytocin, Migraine, Pain, PRLR, OTR, Hormones, Sex differences

## Abstract

Migraine is a severe neurovascular disorder of which the pathophysiology is not yet fully understood. Besides the role of inflammatory mediators that interact with the trigeminovascular system, cyclic fluctuations in sex steroid hormones are involved in the sex dimorphism of migraine attacks. In addition, the pituitary-derived hormone prolactin and the hypothalamic neuropeptide oxytocin have been reported to play a modulating role in migraine and contribute to its sex-dependent differences. The current narrative review explores the relationship between these two hormones and the pathophysiology of migraine. We describe the physiological role of prolactin and oxytocin, its relationship to migraine and pain, and potential therapies targeting these hormones or their receptors.

In summary, oxytocin and prolactin are involved in nociception in opposite ways. Both operate at peripheral and central levels, however, prolactin has a pronociceptive effect, while oxytocin appears to have an antinociceptive effect. Therefore, migraine treatment targeting prolactin should aim to block its effects using prolactin receptor antagonists or monoclonal antibodies specifically acting at migraine-pain related structures. This action should be local in order to avoid a decrease in prolactin levels throughout the body and associated adverse effects. In contrast, treatment targeting oxytocin should enhance its signalling and antinociceptive effects, for example using intranasal administration of oxytocin, or possibly other oxytocin receptor agonists. Interestingly, the prolactin receptor and oxytocin receptor are co-localized with estrogen receptors as well as calcitonin gene-related peptide and its receptor, providing a positive perspective on the possibilities for an adequate pharmacological treatment of these nociceptive pathways. Nevertheless, many questions remain to be answered. More particularly, there is insufficient data on the role of sex hormones in men and the correct dosing according to sex differences, hormonal changes and comorbidities. The above remains a major challenge for future development.

## Introduction

Migraine is a severe neurovascular disorder characterised by recurrent attacks of moderate to severe headache accompanied by nausea, vomiting, and/or photo- and phonophobia. Migraine attacks are aggravated by routine physical activity, which is minimised as far as possible. Patients suffering from migraine with aura will experience reversible neurological symptoms, usually followed by headaches within 60 min. Among them, visual aura is most common, other sensory or speech disturbances are less frequent. Motor or brainstem functions are affected rarely, while retinal aura happens in exceptional cases. Two or more aura symptoms may occur [1]. According to data from the Global Burden of Disease 2019 study, migraine ranks second among causes of disability worldwide and first in females between 15 and 49 years of age [2]. In addition, this disorder highly depends on sex, since migraine is two-to-three times more prevalent in women than in men (21.0% of lifetime prevalence for women and 10.7% for men), especially during the reproductive age [3–5]. In women, the frequency and severity of migraine attacks alter with the fluctuations of sex hormones in cycles and hormonal milestones such as menstruation, pregnancy, lactation and menopause. Migraine has a similar prevalence in the prepubertal period in both sexes but becomes predominant in females along with puberty and the associated hormonal changes [3, 6, 7].

The mechanisms contributing to the pathophysiology of migraine and its sex dimorphism are still poorly understood. Besides the role of inflammatory mediators such as calcitonin gene-related peptide (CGRP) and pituitary adenylate cyclase activating peptide-38 (PACAP-38), which interact with the trigeminovascular system [8, 9] cyclic fluctuations in sex steroid hormones, including estrogen, progesterone and testosterone, are involved in the sex dimorphism in migraine and the genesis of migraine attacks [10, 11]. Nonetheless, it has been reported that pituitary-derived hormones, such as prolactin (PRL) [6, 12], and the hypothalamic neuropeptide oxytocin (OT) [13] may play a modulatory role in migraine and contribute to its sex-dependent differences [12, 14–17]. Likewise, the role of the hypothalamus cannot be overlooked. This area of the brain modulates numerous physiological processes, coordinates behavioural circadian rhythms and regulates the autonomic, cardiovascular, endocrine, and trigeminal pain systems. Altered activation of the hypothalamus, and other regions including the midbrain ventral tegmental area, periaqueductal grey and dorsal pons, is visible from the earliest stages of the migraine attack, in the form of premonitory symptoms such as sleep, mood or appetite disturbances [18]. Twenty-four hours before the attack, the hypothalamic activity in response to trigeminal nociceptive stimulation increases and shows the greatest coupling with the spinal trigeminal nuclei, as determined using a psychophysiological interaction analysis, and measured using functional MRI. This is in contrast to the ictal phase, when the hypothalamus is linked to the rostral pons. This suggests a key role of the hypothalamus as the primary generator of a migraine attack [19]. Potentially, the hypothalamus modulates trigeminal pain, being connected with the trigeminal cervical complex. It appears that changes in signalling, or top-down inhibitory effects on the trigeminal cervical complex, can lead to migraine phase changes [19, 20]. Notably, the hypothalamus is involved in the regulation of the synthesis and secretion of PRL and OT. Paraventricular, supraoptic and accessory magnocellular nuclei of the hypothalamus produce OT in mammals [21]. Moreover, in contrast to the other pituitary hormones, the hypothalamus exerts a tonic inhibition over PRL, primarily via dopamine and other PRL-inhibiting factors [22].

Apart from the influence of sex hormones, functional interactions between central and peripheral regions related to migraine, estrogen receptors, CGRP receptors, OT and oxytocin receptors (OTR) are suggested. Additionally, pituitary peptides, such as PRL, may contribute to the effect of estrogen on migraine [13]. It has been proposed to decrease tonic OT/OTR activity in the premenstrual period, thus lowering thresholds for activation of trigeminal nociceptive afferents. Those changes might be regulated by different factors (such as estrogen level, cholesterol, and IL-6 or other inflammatory mediators) and lead to higher neuronal excitability and migraine attack occurrence [23]. The relationship between PRL and migraine has been investigated for years due to its vascular, electrolyte and fluid-absorbing properties, its effects on dopamine, serotonin (5-HT) and estrogen, and the effectiveness of medications that reduce PRL secretion in the treatment of migraine pain [6, 13–17, 24]. While elevated PRL levels have been observed in almost all migraine patients, this elevation is also associated with chronicity of migraine and worsening prognosis in migraine patients [7, 25]. In recent years, preclinical research on the link between PRL and migraine also improved our understanding of sex-related characteristics of migraine [12, 14–17].

For this reason, the authors of the review decided to investigate the link between the pathophysiology of migraine and two hormones: PRL and OT. Clinical and preclinical studies report a pronociceptive role of PRL as a sensitising factor for pain-related structures also in the trigeminovascular system, which has specific relevance for migraine. On the contrary, OT seems to play an antinociceptive and analgesic role in the trigeminal pain system and the spinal cord, which will be described in more detail in this narrative review.

## Sex hormones and migraine

Depending on the area and the function needed, progesterone and estrogen receptors, which often co-localize, can interact synergistically, antagonistically or neutrally. These different actions are explained through the activation of two estrogen receptors and two progesterone receptors, estrogen receptor α and β (ERα and ERβ) and progesterone receptor A and B (PR-A and PR-B), respectively. Also, a seven-transmembrane-domain receptor named the G protein-coupled estrogen receptor (GPER) that mediates non-genomic estrogen related signalling was found in the pontine nuclei, cerebellum, and spinal trigeminal tract, of which Sp5 is an essential part of the pathway that triggers migraine attacks. Interestingly, its expression is higher in female rat neurons compared to males (65% versus 48% respectively) [26–28]. These estrogen and progesteron receptors modulate the production and metabolic pathways of neurotransmitters and mediators, such as CGRP, 5-HT, glutamate, noradrenalin, nitric oxide and endogenous opioids. Their activation can rapidly modulate vascular tone by producing vasodilating substances, changing the expression of receptors, or changing the activity of ion channels [29]. Significantly higher expression of ERα and ERβ in the female trigeminal ganglion was found, while there was no significant difference in CGRP or PACAP release from trigeminal tissues upon stimulation with potassium chloride in male versus female rats [26]. However, another group has recently found significantly higher CGRP release from the female dura mater upon capsaicin stimulation [30]. Such contradicting findings are not surprising, as it should be taken into account that studies on differences between male and female animals (or humans) highly depend on the hormonal status of the animals at the time of investigation. In recent years, research has focused on the receptors of other sex-related hormones to explain sex dimorphism in migraine. For example, PRL receptor (PRLR) expression was found to be higher in females in the trigeminal ganglion [14, 31]. Moreover, studies on PRL and estrogens have demonstrated that a response to estrogen promotes PRL secretion [32]. In addition, estrogens control PRL expression, PRLR activity and the expression of long isoforms, and PRL-mediated regulation of transient receptor potential vanilloid 1 (TRPV1) channels action in trigeminal sensory neurons [33]. Regarding OT, during menstrual cycles OT levels reflect plasma levels of estrogen. OT production is particularly influenced by the ERβ receptor, while OT receptor expression is influenced by both ERα and ERβ in many brain areas and the trigeminal ganglion [14, 34].

To understand the sex dimorphism in migraine, a careful examination of sex hormones is needed. Sex hormones are classified into three categories: hypothalamic, pituitary and gonadal. The hypothalamic-pituitary–gonadal pathway becomes activated by an increase in estrogen, androgen and progesterone synthesis in gonads, and initiates puberty [7]. The menstrual cycle in women in their reproductive phase begins with menses and ends on the last day before the next menstruation. This rhythmic change requires harmonised hypothalamic (luteinizing hormone-releasing hormone, gonadotropin-releasing hormone), pituitary [luteinizing hormone (LH), follicle-stimulating hormone (FSH)] and ovary (estrogen, progesterone) activity [35]. In a substantial number of migraine patients, the number of migraine headaches increases during the menstruation (also called menstruation-related migraine attacks), coinciding with a cyclical rapid decrease in estrogen, progesterone, and OT levels, which precedes the onset of menstrual phase. Due to these observations, and uncertainty about the estrogen withdrawal hypothesis as a key role in the pathogenesis of migraine, the role of OT as a potential (anti)migraine hormone is emphasised [7, 13]. Moreover, PRL levels increase at the end of the follicular phase during the menstrual cycle [36]. One study found that PRL levels were higher in patients during menstrual migraine, although this was not significantly different from controls [25]. Additional studies including a higher number of patients should further investigate the levels of PRL during menstrual migraine.

The link between menstruation and migraine is significant, as it affects up to two-thirds of women who suffer from migraine [34, 37]. Both migraine without aura (MO) and migraine with aura (MA) can occur during a menstrual migraine attack, although the first type seems to predominate [38, 39]. Pregnancy is a period when cyclic hormone fluctuations disappear, however, sex hormones such as estrogen and progesterone, as well as OT, PRL, beta human chorionic gonadotrophic hormone (β-HCG) and relaxin increase. Reproductive hormones that increase during pregnancy drop with childbirth and return to normal levels within six weeks in the postpartum period, with the exception of PRL in breastfeeding women [35]. Menopause signifies the end of the reproductive capacity of women, and is manifested by a period of 12 months amenorrhoea without any other explanation. After menopause, laboratory tests reveal high levels of FSH and LH and low levels of estradiol and progesterone [40, 41]. Also, caused by estrogen loss, blood OT levels decrease gradually, which affects sexual ability and vagal activity [24, 42]. PRL levels seem to be higher in premenopausal women in comparison to postmenopausal women and men. This is consistent with the changes, or more precisely, the decrease of menopausal hormones affecting PRL secretion (prolactin releasing factors), such as OT, estrogen and thyrotropin-releasing hormone [43]. Due to the decrease in female hormones, symptoms such as sleep disorders or osteoporosis are observed; however, hormone replacement therapy may be used to counter these negative effects [41]. In the postmenopausal period, the prevalence of MO decreases as the time since the last menstrual period increases. Such a relationship is not observed for MA. The change in the nature of this type of migraine is rather associated with the increasing age of the patient [44].

Both MO and MA are modulated by reproductive life changes in women, but critical female hormonal changes seem to affect it differently. Therefore they can be treated as two distinct nosological entities [45]. Current reports see a fall in plasma estrogen levels as a trigger of MO attacks. In this type of migraine, the greatest risk of an attack is in the late luteal (premenstrual) phase, while it decreases with higher estrogen levels. Similar observations were done in pregnant women suffering from MO [46]. The development of a normal pregnancy is accompanied by a steady increase of endogenous blood levels of estrogen and progesterone. Those changes are responsible for the gradual improvement of MO symptoms, with the percentage of improvement increasing with the duration of pregnancy [47, 48]. As underlying mechanisms of MA, increased susceptibility to cortical spreading depression (CSD) was detected in animal studies of familial hemiplegic migraine (FHM) type 1 (according to ICHD-3, FHM is a subtype of MA [1]). Estrogen increases the susceptibility to CSD and with increased levels of this hormone, the brain becomes more susceptible to trigger factors [7, 49]. This could be the reason why MA may worsen during pregnancy, contraception or hormonal replacement use, but also throughout a normal menstrual cycle when levels of estrogen are elevated [50]. Interestingly, since estrogen and androgen have a reciprocal influence on CSD-susceptibility, male mouse models of FHM type 1 showed reduced receptivity due to testosterone. Androgens decrease severity and attack frequency in migraine (and perhaps the risk of MA [51]), suggesting the possibility of androgen treatment for men and women [7, 52, 53]. In non-obese males with migraine, higher levels of estradiol with relative androgen deficiency was reported; however, the cohort was too small to draw conclusions and future studies are needed [54]. Nonetheless, the potential effect of testosterone (converted to estrogen) was investigated in animal models [55]. In females, as mentioned earlier, testosterone may play a protective role demonstrated as improvement of headache intensity. Perhaps, this can be explained by the inhibition of CSD by testosterone and/or anti-nociceptive and anti-inflammatory properties of this hormone [43]; however, this is beyond the scope of this review.

The fact that migraine prevalence is three times higher in women than men raises the question whether sex hormones may be involved in migraine generation. The presence of headaches has been studied in male to female transsexuals. They used antiandrogens to suppress male sex characteristics and estrogens to induce female sex characteristics. In a Dutch population, the migraine prevalence of 26% in male to female transsexuals is similar to that of 25% in genetic females, and significantly greater than the prevalence of 7.5% in men [2, 56]. These findings could propose an estrogen-mediated regulation of pain and subsequent OT- and/or PRL-involved mechanisms in migraine [56]. The potential effect of hormone therapy on headache disorders also seems significant in transgender patients, however, little data is available and further research is needed [57, 58].

## Prolactin

Prolactin (PRL) is a 23-kDa helical protein produced and secreted primarily by the lactotroph cells of the anterior pituitary gland, but is also secreted from many diverse non-hypophyseal peripheral areas such as deciduoma tissue, mammary gland, ovaries, prostate, testes, endothelium, lymph nodes, skin, adipose tissue, inner ear cochlea, and immune cells. Besides the classical role of promoting and maintaining lactation, its biological actions are not limited only to reproduction. PRL can modulate many unrelated functions in both females and males and plays a role in the immune response, brain and behaviour, osmoregulation, growth and metabolism [59–61]. PRL can mediate effects in a large variety of cells of the immune system including monocytes, neutrophils, macrophages, lymphocytes, natural killer cells, and microglia. In the brain, it exerts inflammatory and anti-inflammatory effects, depending on the cytokines that it interacts with [62]. Moreover, PRL has antiangiogenic, vasoconstrictive and anti-vasopermeability actions and blocks blood vessel growth and dilation [63]. The diversity of actions of PRL stems from the fact that it activates different intracellular signalling pathways, resulting in heterogeneity of target genes [64, 65]. The PRL gene is localised on chromosome six and composed of five exons and four introns [66]. The transcription is regulated by two independent promoter regions of which the proximal one directs PRL expression in the pituitary, while the distal promoter controls extrapituitary sites of expression [67]. In line with the functional diversity, the biological actions of PRL are mediated by the PRLR which is expressed in different target tissues and has also been identified in the brain [64]. This receptor is a single membrane-bound protein and a member of the class I cytokine receptor superfamily that includes the growth hormone (GH) receptor, leptin receptor, erythropoietin receptors, and receptors for many other interleukins [67]. The PRLR gene is located on chromosome 5 and consists of 11 exons, which make up the 5′untranslated region and the coding one. The 5′UTR contains exon 1, which exists in five alternative forms characterised by tissue-specific activity. The transcription of the exon 1 is performed by three different promoters (PI, PII, PIII). PI is active in gonads, PII is specific for the liver and PIII is active in all PRL sensitive tissues [66]. This leads to the expression of different isoforms of the receptor. In particular, all of them share a common extracellular and transmembrane domain and a variable intracellular one, which can be short, intermediate or long and determines the differences in the signal transmission pathways [68]. In humans, five isoforms of the receptor have been described: the long form (PRLR-L), the intermediate form (PRLR-I), and three short isoforms (PRLR-S) ΔS1, S1a and S1b [16]. The active complex consists of a single ligand molecule and two receptor molecules, each containing an extracellular, transmembrane and intracellular domain containing tyrosine residues that determine phosphorylation which follows receptor activation [61]. PRL binds to two extracellular interaction sites with different affinities called binding domain 1 and binding domain 2, which induce dimerization of the two receptor molecules. The intracellular domain consists of two main regions, Box 1, which is required for the JAK2 activation, and Box 2 which is phosphorylated by JAK2 and acts a key role for the binding and activation of numerous proteins. This leads to the activation of different downstream signalling pathways which can produce different cell responses and explains the versatility of the actions of PRL [66, 69, 70]. There are three main routes, through which PRL can induce genomic responses: mitogen-activated protein kinase (MAPK) cascade, signal transducer and activator of transcription (STAT) and the phosphoinositide 3-kinase (PI3K) pathway [16]. Activation of the long isoform of PRL-R causes the phosphorylation of STATs protein, by JAK2 molecules and its translocation to the nucleus, activating γ-interferon activation sequence (GAS) on target genes [66]. The tyrosine residues of the short isoform cause the activation of the MAPK and PI3K cascade, which are involved in the activation of a wide range of transcription factors involved in cell proliferation and mediate some of the antiapoptotic action of PRL [61, 71>].

Physiological concentrations of PRL in adults are 10–25 μg/l in women and 10–20 μg/l in men and they are closely regulated by a circadian rhythm [72]. There is an increase in the amplitude of the PRL secretory pulses that begins about 60–90 min after sleep onset. The lowest PRL concentrations are found during REM sleep and the highest concentrations are found during non-REM sleep. Moreover, diurnal variation of PRL secretion is not an inherent rhythm but depends on the occurrence of sleep [73]. It has also been shown that sleep deprivation can induce a reduction in PRL levels especially in the second part of the night [74]. Nevertheless, there is variability in basal PRL levels across different external environmental stimuli and physiologic or pathologic internal stimuli. Changes in PRL secretion are induced by hypothalamic inhibitory or stimulatory hormones that act on lactotroph cells via the hypothalamic-pituitary portal circulation. The neuroendocrine neurons of the hypothalamic regulatory circuit can produce PRL inhibiting factors such as dopamine, somatostatin, and gamma-aminobutyric acid (GABA), or PRL releasing factors, such as thyrotropin releasing hormone (TRH), OT, and neurotensin [71]. The balance between the two signals determines the amount of PRL released from the anterior pituitary gland. The main inhibitory signal is mediated by the neurotransmitter dopamine, which acts on dopamine receptors (D_2_) on the surface of lactotroph cells. On the other hand, the primary stimulus signal is due to TRH, which binds to type 1 TRH receptors expressed in both thyrotrophs and lactotrophs [59, 73, 75, 76]. Estrogen is also an essential physiological activator of PRL synthesis. Its action is mediated through binding to the estrogen response element (ERE), which is located within the distal enhancer of the PRL gene and results in its transcription. This also explains why the inhibitory action of dopamine is partially blocked by estrogen [73]. Unlike other pituitary hormones, the hypothalamus exerts a predominantly inhibitory influence on PRL secretion because lactotrophs appear to spontaneously secrete this hormone. For this reason, the hypothalamus provides inhibitory rather than stimulatory control [68]. Furthermore, circulatory PRL levels seem to have a negative feedback on their own secretion, called short-loop feedback or autofeedback. In this way, PRL itself stimulates hypothalamic dopamine secretion via PRLRs located on hypothalamic neurons [77]. Recent studies have shown that PRL secretion seems to be coupled in some way to the pattern of gonadotropin secretion. Activation of kisspeptin neurons in the arcuate nucleus drives the pulsatile release of gonadotropin-releasing hormone (GnRH) and consequently the pulses of LH secretion from the pituitary gland. At the same time, kisspeptin can stimulate PRL secretion, through the suppression of tuberoinfundibular dopamine release. As a consequence, the hypothesis of a single “pulse generator” seems feasible, in which each episode of kisspeptin release driving an LH pulse may also cause a temporally-linked pulse of PRL secretion [77].

## Role of prolactin in migraine pain

Besides playing an essential role in several physiological processes, PRL is associated with different pain conditions, including: postoperative [78], inflammatory [14, 79], neuropathic [14] and orofacial pain [80], as well as primary (i.e. migraine and cluster headache) and secondary headaches (i.e. prolactinoma- and/or pituitary diseases-associated headaches) [6, 12, 14–17]. This association may be based (among others) on: (i) the central and peripheral (in)direct effects of PRL by stimulating the immune system and neurons involved in nociception [78, 79]; (ii) the evidence of high levels and increased release of both endogenous pituitary and extra-pituitary PRL during pain [78, 80]; and (iii) the expression of PRLR in pain-related structures [12, 14, 17, 31, 81].

Regarding the relationship between PRL signalling and migraine (or prolactinoma- and/or pituitary diseases-associated headaches), clinical and preclinical studies have suggested the involvement of PRL and PRLR in headache disorders. In this respect, preclinical studies have demonstrated that PRL contributes to migraine pathogenesis involving sex-specific mechanisms, which may help to understand the sex-related differences in migraine. These findings have been supported considering that: (i) dural administration of PRL produces long-lasting migraine-like behaviour responses in female but not in male rodents [15]; (ii) administration of dopamine D_2_ agonists like bromocriptine [12] or cabergoline [17] results in decreases in serum PRL levels only in female mice; (iii) PRLR is expressed in both trigeminal ganglion sensory neurons [12, 17, 31] and in neuronal fibres that innervate the dura mater [15], showing a higher expression in female than in male mice; (iv) the co-localization of PRLR and calcitonin gene-related peptide (CGRP) in sensory nerves [14, 15, 31]; and (v) there is a crosstalk between PRL, CGRPergic and serotonergic systems [15, 17]. In this respect, PRL promotes increases in CGRP release in female but not in male rodents, inducing female-specific migraine-like behavioural responses [15, 82], which decrease in the presence of CGRP_8-37_, a CGRP receptor antagonist [15]. Moreover, in an animal model of medication-overuse headache induced by repeated administration of sumatriptan [17]: (i) increases in serum PRL levels were observed only in females; (ii) co-administration of sumatriptan plus cabergoline prevents allodynia and downregulation of the short isoform of PRLR, which is involved in nociception; and (iii) PRLR co-localizes with the 5-HT receptors (i.e. 5-HT_1B/1D_ receptors, the main therapeutic targets for triptans in the acute treatment for migraine) [17]. Likewise, PRL release is modulated by 5-HT [83]. It has been demonstrated that female MO patients have a significantly higher rise in serum PRL levels from baseline than controls in response to the administration of buspirone, a 5-HT_1A_ receptor agonist, showing higher sensitive central amine receptor function [84, 85]. Furthermore, other neuropeptides considered as migraine triggers, such as PACAP-38, induce effects on the PRL system. PACAP-38 is a multifunctional neuropeptide with strong vasodilatory action [86], which is involved in the regulation of hypothalamus-pituitary axis [87]. In fact, it has been shown that PACAP-38 infusion increases PRL release in MO patients [88] and in rat pituitary somatolactotroph GH3 cells [89].

Evidently, the mechanisms involved in sex dimorphism in pain, including migraine, are not yet fully characterised. Nevertheless, they could be explained also by an (in)direct dysregulation in PRL signalling (i.e. central and peripheral mechanisms) involving a higher expression of PRLR in sensory nerves and nociceptor sensitization in females [14–17, 78, 81], which may be related to the higher risk and the increase in the prevalence, recurrence, and severity or chronification of pain and migraine in females.

### Prolactin system in the modulation of neuronal excitability

Transmission of nociceptive information from peripheral nociceptive neurons to second-order interneurons in the spinal cord affects the perception of pain. The spinal cord dorsal horn can be considered as a critical place for the transfer of this information [90]. Changes of this transmission due to inflammation, injury or activity, may lead to abnormal signalling in nociceptive pathways. Sensitization manifests as psychophysical changes such as lowered pain threshold, spontaneous stimulus-independent pain, or increased response to suprathreshold stimuli, and plays a role in pain signalling. In this respect, activation of both peripheral nociceptors and central generators (spinal or supraspinal) can trigger an increase in sensitivity and excitability of neurons, resulting in enhanced nociceptive signalling and pathological pain perception (i.e. allodynia and hyperalgesia) [91–94]. In migraine, central sensitization triggers hyperexcitability of trigeminovascular neurons, leading to the development of migraine attacks [95]. This excitability, specifically in the neocortical neurons, is responsible for the transition to cortical spreading depression, which is regarded as the underlying mechanism of aura and a trigger of headache attacks [96].

The increased neuronal excitability in nociceptive pathways can be regulated by several factors including steroids and pituitary-derived hormones such as PRL [14, 94, 97–99]. In this respect, PRL is capable of inducing action potential firing in sensory nerves, which can be regulated via the activation of PRLR [14, 99]. Nevertheless, although PRLR is expressed in both males and females, PRL-induced neuronal excitability in inflammatory pain was detected only in dorsal root ganglion neurons of female mice [14, 99], which may be due to a higher expression of the PRLR in females. Therefore, it seems that PRL-regulated sensory neuronal excitability is a female-specific mechanism which also depends on estrogen [14]. Furthermore, PRL can regulate excitability in different neuronal circuits including the magnocellular neurons of the supraoptic and paraventricular nuclei [100] and the tuberoinfundibular dopamine neurons [101] via the PRL-induced release of OT or dopamine, respectively, as a consequence of the neuronal activation by PRL, generation of action potentials and increased calcium influx [99–101].

### Prolactin signalling modulates TRP channels

As mentioned above, one of the possible associations between PRL and pain involve (in)direct modulatory effects of PRL by stimulating sensory neurons and producing nociceptor sensitization [78, 79]. In fact, it has been demonstrated that sensitization of TRP channels is one of the mechanisms involved in the modulation of inflammatory pain by activation of PRL signalling [33, 99, 102].

TRP channels, specifically those belonging to the vanilloid (i.e. TRPV1), melastatin (i.e. TRPM8) and ankyrin (i.e. TRPA1) subfamilies are associated with pain, including migraine [103, 104]. The peripheral modulation of pain by PRL signalling via TRP channels is based on the facts that: (i) PRLRs co-express with TRPV1 channels in sensory nerves [33, 102]; and (ii) exogenous PRL induces sensitization of TRPV1, TRPM8 and TRPA1 channels [33, 79, 102, 105] via endocrine, autocrine and/or paracrine pathways on sensory nerves [80]. This sensitization involves the activation of the short isoform of PRLR and protein kinase C-delta/phosphatidylinositol 3’-kinase (PKCδ/PI3K) pathways [79, 105]. In this respect, activation of the TRPV1, TRPM8 and TRPA1 channels by capsaicin, mustard oil or menthol, respectively, induced a significant potentiation of calcium influx after PRL pretreatment in mice dorsal root ganglion neurons [79, 105] and in rat trigeminal ganglion neurons [33]. This PRL-induced sensitization of TRP channels is a sex-dependent mechanism considering that the capsaicin-, mustard oil- or menthol-evoked increased calcium influx was observed only in neurons of female mice, with no effect on neurons of male mice [79]. In addition, behavioural animal studies have shown that PRL can induce nociception in female rats at proestrous but not in ovariectomized rats [33], which suggest that PRL-induced nociception effects are estrogen dependent [14, 33]. Likewise, the activity of PRL and the expression of PRLR isoforms, particularly the long isoform in trigeminal sensory neurons, is upregulated by estrogens. This results in signalling pathway activation and nociceptor sensitization through increased phosphorylation of TRPV1 channels [33].

In conclusion, there is clear evidence that PRL signalling plays an important role in pain pathways, including migraine. Considering that PRL and PRLR display a sex-dependent activity, PRL signalling might represent an important approach in understanding sexual dimorphism in the pathophysiology of pain and migraine. Nevertheless, future research is needed to elucidate the specific mechanism involved in the sex-specific activity of PRL in pain modulation in order to investigate whether targeting PRL signalling could represent a potential therapeutic approach for pain disorders, including migraine.

## Prolactin levels in migraine patients

Increased serum PRL levels have been described in patients with headaches, especially migraine [106, 107]. A meta-analysis from 2019 showed higher PRL levels in the migraine patient group compared to controls based on 13 studies, although there was a high degree of heterogeneity among studies [16, 107]. In comparison, a few studies showed that reduced PRL levels accompany migraine attacks [108, 109]. One study reported decreased PRL levels in 20 male patients with migraine as compared to 20 male controls with non-migraine headaches [108]. Masoud et al. showed a decrease in PRL levels during headache attacks as compared to the interictal phase [109]. Interestingly, this decrease was more prominent in migraine patients compared to patients with non-migraine headaches [109].

In this respect, clinical studies have shown that high levels of PRL are associated with the progression of migraine [106, 110], therefore, PRL is considered as a worsening factor for migraine [106]. Likewise, patients with prolactinoma-associated headaches, which can involve different headache phenotypes (including cluster headache, trigeminal autonomic cephalalgias, short-lasting unilateral neuralgiform headache, and migraine with visual aura), also present with high serum levels of PRL [111–116]. These increased levels of PRL can be related to dysregulation of the hypothalamic-pituitary axis [117–119] and may contribute to the onset of migraine and/or migraine-like headache attacks [114]. Furthermore, this relationship is supported by the suppression of PRL release from the pituitary gland after treatment with dopamine D_2_ and D_3_ agonists, such as bromocriptine [112, 114, 120] or cabergoline [111, 112, 114] in patients with prolactinoma-associated headaches. These treatments lead to a reduction of the frequency of headache attacks, probably due to a reduction in tumour size, normalisation of serum PRL levels, and/or blockade of nociceptor sensitization [111]. Thus, there seems to be an evident link between blood PRL levels and migraine. However, further research is warranted on the directionality of PRL alterations in migraine and whether an interplay of both decreased and increased PRL levels plays a role in migraine. Moreover, it is not clear yet how altered PRL levels may influence different headaches (i.e., episodic and chronic migraine, tension-type headache, cluster headache) and possibly aura or prodromal symptoms [16].

The link between migraine and PRL also becomes evident when taking into account that the normalisation of PRL levels can alleviate headache (i.e., through PRL-decreasing medication, such as 0.5 mg of cabergoline twice a week) [106, 107, 121, 122]. Furthermore, studies investigating serum PRL levels in patients with chronic migraine (CM) found increased PRL levels in CM [106]. In addition, one study found lower nocturnal PRL peaks in patients with CM compared to healthy controls [123]. Thus, elevated PRL levels may potentially worsen migraine symptoms or lead to migraine chronification [106, 124, 125].

Another option to study the link between PRL and headache is by investigating hyperprolactinemia, a condition characterised by elevated serum PRL levels of more than 0.1–0.2 mg/l [126]. Hyperprolactinemia can occur due to prolactinomas [127–129] but can also have other causes like comorbidities (i.e., renal failure, hypothyroidism), medication, stress or pregnancy [127, 129]. Prolactinoma-related headaches are common in 37–83% of prolactinoma cases [124, 130] and migraine-like headaches have been described in hyperprolactinemia [112, 114, 120, 121, 124]. In addition, other headache disorders may be present, like paroxysmal hemicrania, short-lasting unilateral neuralgiform headache with conjunctival injection and tearing (SUNCT) syndrome, or cluster-like headache [122, 131, 132]. While it was postulated in the past that tumour mass and pressure on nerves may cause secondary headaches [112, 130], it was recently shown that headache symptoms do not depend on tumour size [112, 133]. Instead, the authors indicated that PRL may exert a modulatory effect on the neuronal excitability rather than tumour size [99]. Moreover, hyperprolactinemia was found to worsen migraine attacks [106, 114]. Nevertheless, it should be noted that migraine-like headaches associated with hyperprolactinemia are often unresponsive to common preventive medication for migraine and only resolve after normalisation of PRL levels [121].

## Effect of current (anti-migraine) treatment on prolactin levels

### Effect of acutely acting anti-migraine treatment on PRL levels

Pharmacological treatment of migraine is divided into two groups: the treatment of the acute episode of pain, and preventive or prophylactic treatment. Controversy exists on the effects of migraine treatments on PRL levels (Table 1). First, the influence of acute anti-migraine treatment on PRL levels is discussed. Common pain relievers, such as aspirin, acetaminophen, and nonsteroidal anti-inflammatory drugs (NSAIDs), can alter levels of sex hormones and PRL.

**Table 1:** Influence on prolactin levels of approved treatments for migraine (acute and prophylaxis)

	**Treatment**	**Prolactin levels**
**Acute**	Acetaminophen	↓ Decrease [134]
	Aspirin	No evidence [134]
	Triptans (sumatriptan)	↓ Decrease [135, 136]
**Prophylactic**	Propranolol	↓ Decrease – chronic use [137, 138]
	Flunarizine	↑ Increase – transient effect [139, 140]
	Amitriptyline	No effect [141–143]
	Topiramate	No evidence [144–146]
	Valproate	↑ Increase [146–148]
	Botulinum toxin	No evidence [149, 150]

An inverse relationship between the frequency of paracetamol consumption and PRL levels has been described. PRL levels were lower in women who used acetaminophen. Regarding the duration of the treatment, the duration of acetaminophen use was also inversely related with the levels of free testosterone and DHEAS. Duration of acetaminophen use was not associated with PRL [134]. There is some evidence on the influence of aspirin and the variation of hormone levels, specifically, the levels of estradiol, estrone, estradiol/testosterone ratio and PRL. Longer duration of aspirin use was associated with higher follicular estrone levels. Also, the frequency of aspirin use was positively associated with follicular estrone and follicular free estradiol, and inversely associated with DHEAS. These effects vary according to the body mass index (BMI), but do not fluctuate depending on age. Among women with a BMI higher than 25, frequent use of aspirin was inversely associated with luteal estradiol. There are no studies that correlate the use of NSAIDs with the modification of PRL levels [134].

Triptans are drugs of first choice for the treatment of migraine attacks with moderate to severe intensity. They are serotonergic receptor agonists (5-HT_1B/1D_) [135]. There is evidence of decreased PRL levels after the use of sumatriptan [135]. Sumatriptan is a specific 5-HT_1B/1D/1F_ serotonergic receptor agonist, with low affinity for 5-HT_1A_, 5-HT_5A_ and 5-HT_7A_ receptors [151]. Evidence from preclinical studies suggests that GH release may be mediated by both 5-HT_1B_ and 5-HT_1D_ receptors. Sumatriptan significantly increases GH levels and inhibits PRL release. The ability of a 5-HT receptor agonist to lower plasma levels of PRL creates some controversy. 5-HT_1A_ receptor agonists have been found to increase PRL levels. If sumatriptan has the ability to act on 5-HT_1D_ receptors, which are present both presynaptically and postsynaptically, they could decrease 5-HT levels and thus decrease PRL levels, and finally increase GH levels. In human studies, the dynamics of PRL values after administration of sumatriptan versus placebo were studied. After the administration of sumatriptan, the plasma levels of PRL decreased significantly, after 30 min of administration and in the rest of the measurements [135, 136].

### Effect of prophylactic anti-migraine treatment on PRL levels

There are various treatments approved as preventive/prophylactic in migraine. The most commonly used drugs are: antihypertensives, calcium channel blockers, antidepressants, antiepileptics and botulinum toxin. Within the group of antihypertensives, beta-blockers are most frequently used. There are studies that demonstrate a reduction of PRL levels after the use of propranolol [137]. Chronic use of propranolol (more than 6 weeks) produces a significant decrease in nocturnal PRL concentrations in healthy controls [137, 138]. Although there is controversy about the mechanism by which propranolol decreases PRL levels, it may be partially due to a central mechanism, or in relation to the antihypertensive effect of the drug. The chronic use of propranolol may decrease the levels of LH, but FH and testosterone levels decrease after a single dose of propranolol. Human studies in males showed no acute effect of propranolol on the pulses of LH [138].

Flunarizine is a calcium channel blocker commonly used in the prophylactic treatment of migraine. Apart from blocking calcium channels, it also has an antagonistic effect on histaminergic H1 receptors, which could affect hormone secretion [139]. Evidence of an effect of this drug on PRL levels is controversial. A transiently significant increase in PRL levels after the administration of flunarizine was demonstrated but this effect was not maintained after 90 days of treatment [139, 140]. It is possible that the transient increase in PRL levels is due to its action on calcium channels, as well as the antidopaminergic effect [140].

Tricyclic antidepressants are approved as preventive treatment for migraine, and within this group the most widely used is amitriptyline [141]. The hormonal effect of amitriptyline varies in relation to the history of depression of the patient. In patients with depression, higher PRL levels have been reported after the use of amitriptyline, while this was not observed in healthy patients [142]. Both single administration and chronic treatment are not associated with changes in the levels of this hormone [141–143].

Another group of drugs used in the prophylaxis of migraine are the antiepileptics, among them are approved: topiramate and valproic acid. Both are broad-spectrum anti-seizure drugs. Topiramate acts as an antagonist of glutamatergic receptors (AMPA and kainate), enhancing the activity of GABA_A_ receptors and inhibiting sodium channels [144]. There is a study comparing the effects of topiramate with flunarizine on PRL levels in migraine patients, with no evidence of hormonal changes after use of the two drugs [145]. Valproic acid shares a mechanism of action with topiramate, being a GABAergic drug; although it has a more complex mechanism of action, modulating the conductance of calcium, potassium, and sodium [146]. Regarding the neuroendocrine effects of valproic acid, studies in humans found evidence of increased PRL levels. The effect is due to the modification of the GABAergic, noradrenergic, and serotonergic tone that modulate the release of dopamine [147, 148].

Finally, in relation to botulinum toxin, a study has been carried out in female animal models to determine the influence on the levels of sex hormones (FSH, LH and progesterone). A decrease in FSH and LH levels was observed, with an increase in progesterone levels [149]. There is no clear evidence on the effect of botulinum toxin on PRL secretion. Although there is a study that suggests the inhibitory effect of a botulinum toxin derivative on the growth hormone–insulin-like growth factor-I axis (GH/IGF1 axis). In in vitro models, a decrease in GH synthesis was observed associated with an increase in PRL levels [150].

### Effect of PRL level modifying drugs

Just as some treatments currently used for migraine can increase PRL levels, there are other treatments aiming at modifying the level of this hormone as the primary target (Table 2). Before discussing the effect of individual drugs on PRL levels, it is important to understand the physiological pathways of PRL regulation. The hypothalamus exerts a tonic inhibition on PRL, the agents responsible for this inhibition are prolactin inhibiting factors dopamine, histamine (acting at the H2-receptors) and acetylcholine. Also, GABAergic receptors are involved in an inhibitory control of PRL. Additionally, the hypothalamus contains substances which can promote the release of PRL, such as polypeptide PRL-releasing factor, 5-HT, melatonin and histamine (acting at the H1-receptors) [22].

**Table 2:** Treatments that can modify levels of prolactin

Treatment	Mechanism of action	Prolactin levels
Bromocriptine	Dopamine direct-acting receptor agonist	↓ Decrease [22, 114]
Chlorpromazine	Dopamine receptor antagonist	↑ Increase [22, 152]
Methysergide	Competitive blocker of serotonin receptors	↑ Increase – transient effect [22, 153, 154]
Fenfluramine	5-HT_2B_ receptor agonist	↑ Increase [22, 155]

The group of PRL-lowering drugs includes direct acting dopamine agonist (e.g., dopamine, apomorphine), indirect acting dopamine agonist (e.g., methylphenidate, amphetamine), drugs that impair serotonergic neurotransmission (e.g., methysergide), gamma-aminobutytic acid mimetic drugs (e.g., sodium valproate), histamine H2-receptors agonist and cholinergic receptor agonist. Major prolactin-stimulating agents are dopamine receptor antagonists (atypical antipsychotic drugs), drugs capable of central nervous system dopamine function (carbidopa/benserazide), drugs enhancing serotonergic neurotransmission, 5-HT reuptake blockers, H1-receptors agonist and H2-receptors antagonists [22, 152, 153, 156].

The dopamine mimetic drugs are classified as direct and indirect-acting receptor agonists. Bromocriptine is the prototype of the direct-acting receptor agonists. These drugs act either at the dendrites or the soma of hypothalamic neurons secreting PRL inhibiting factors or by stimulating dopamine in the anterior pituitary gland. One disadvantage of this group is the ability to affect both central and peripheral 5-HT and noradrenaline receptors [22]. Historically, bromocriptine has been used cyclically and continuously in patients with menstrual migraine in a small number of studies, with a statistically significant reduction in migraine attacks. Studies have shown that the use of bromocriptine in patients with prolactinoma significantly reduces migraine-like headache attacks, and some patients are even completely pain-free [114].

Chlorpromazine is an antipsychotic drug that acts as a dopamine receptor antagonist and blocks noradrenaline receptors. The dopamine receptor blockade by antipsychotic drugs increases PRL secretion in animals and humans. This effect is due to direct blockade of dopamine receptors on pituitary lactotrophs through the hypophyseal portal vessels [22, 152].

Methysergide is a competitive antagonist of 5-HT receptors, but the exact mechanism of action on PRL release remains controversial. Some studies suggest that a dopaminergic inhibitor pathway is involved [153]. In studies conducted in rats, an increase in PRL levels occurred shortly after the parenteral administration of methysergide, while after one hour no effect or decreased levels of PRL were observed. The acute rise may be caused by antagonism at the dopamine receptors [22]. For migraine, the clinical effect was generally excellent, but it was later found to cause retroperitoneal fibrosis after chronic intake [154].

As we previously discussed, drugs that can increase the availability of 5-HT at postsynaptic sites have PRL-releasing properties. Fenfluramine can release 5-HT from presynaptic terminals and activate 5-HT transmission. However, high doses of fenfluramine can also block dopamine receptors [22, 156]. Fenfluramine can be a migraine attack inducer [155].

## Prolactin, migraine and males

PRL is produced by the anterior pituitary gland and mainly involved in maternal behaviour [157, 158], however, it is also present in the male pituitary and studies indicate the presence of PRLR in the male reproductive organs [157–160]. PRL increases steroidogenesis by modulating the release of gonadotropins from the anterior pituitary gland by increasing the number of LH receptors in Leydig cells in the testicles. In addition, PRL increases the number of FSH receptors in Sertoli cells. In germ cells, it regulates the transformation of spermatocytes and spermatids [161–164]. Therefore, PRL has an important role in fertility in males [165–169]. In addition, PRL has a role in paternal behaviour [170, 171]: men with high PRL levels allocate more time to their children for playing, are more sensitive to the crying of their children [170], and more sensitive and interested in their babies [171].

PRL may have a role in mechanisms related to pain as well as reproduction, fertility, and paternal behaviour [79]. Moreover, PRL levels can be elevated in stressful situations in both males and females [12], and PRL was shown to be upregulated in inflammatory and post-operative conditions in both sexes [79]. PRLR is expressed in trigeminal ganglion sensory neurons and fibers innervating the dura mater in both male and female mice, albeit with a higher expression in females [12, 15, 31]. In addition, after dural administration of PRL, migraine behaviour occurs in female mice, but is absent in male mice [15]. Moreover, PRL can increase the release of CGRP in the female dura, but it does not have the same effect in the male dura, suggesting that the nociceptive effect of PRL may be limited to females [15]. Further studies support the concept of a female-specific mechanism that links PRL and migraine [17, 33, 81]. In this respect, sumatriptan administration was shown to increase PRL levels in female mice, while no effect was seen on PRL levels in males. Moreover, inhibition of circulating PRL levels using cabergoline prevented cutaneous allodynia in females but not males [17]. Furthermore, ​​ 5-HT_1B_ and 5-HT_1D_ receptors co-localize with the PRLR in the trigeminal ganglion, mainly in females, and re-administration of sumatriptan down-regulates the TGV1 PRLR-L isoform in female mice only, with no change in PRLR-S. Based on these results, they reported that PRL levels and PRLR signals are increased in females in a mouse model for medication overuse headache, suggesting a potential role of the hypothalamus and the neuroendocrine system in the chronicity of migraine, especially in females [17]. Furthermore, it has been reported that the PRL response is higher in females compared to male sensory neurons in the dorsal root ganglion and trigeminal ganglion [31, 79, 105, 172]. However, Patil et al. stated that PRLR-L and PRLR-S mRNA expression in dorsal root ganglion neurons is not dependent on sex [31].

Overall, the preclinical studies point to a female-specific mechanism of PRL in migraine. However, human data is not sufficient to be able to draw conclusions on the effects in men and studies on PRLR expression in pain structures in men do not provide sufficient information. Interestingly, Li et al. found higher PRL levels in male migraine sufferers compared to healthy men, just as female migraine sufferers had higher PRL levels compared to healthy women [107, 118], suggesting that also in men the PRL system could be affected during migraine. Therefore, additional studies are needed to reach a conclusion about whether PRL could be an effective target in male migraine patients.

## Oxytocin

OT is a peptide hormone, or neuropeptide, mainly produced in the magnocellular neurosecretory cells of the hypothalamus, specifically in the paraventricular, supraoptic and accessory magnocellular nucleus. From these nuclei, OT is further transported from neurosecretory granules along axons within the hypothalamo-neurohypophysial tract to axon terminals in the posterior pituitary. OT is stored in Herring bodies from which it is released to the circulation with effect on different tissues through binding to the OTR [21, 173]. OTRs are expressed in the peripheral system, for instance in the uterus or breast, as well as in the central nervous system. Their plasticity is strongly influenced by the levels of estrogen, cholesterol and corticosteroids but also by exogenous substances, such as fluoxetine and cocaine [174]. Via peripheral release from the posterior pituitary into systemic circulation, OT is involved in lactation and parturition, as well as the regulation of social behaviour [175, 176]. Furthermore, this neuropeptide is dendritically released by hypothalamic neurons and can passively diffuse into various brain structures. However, a sufficient amount of OT is required to activate the OTR, which can be accomplished directly via long-range axonal projections of hypothalamic OT neurons [177, 178]. The limbic regions and the brain stem receive direct innervation by OT fibers. Nonetheless, the phenomenon of local OT release has also been identified in areas such as the amygdala, with a particular association with stressful situations and with the reward system [179]. Additionally, OT reduces GABAergic inhibition in synapses (especially in the hippocampus, auditory cortex, piriform cortex, and paraventricular neurons) [176, 180]. In recent years, reports indicate the analgesic and antidepressant effects of OT [181]. According to human and animal models, OT is linked to nociception and pain, and this phenomenon can be explained through both physiological and psychological mechanisms. It has also been suggested that oxytocinergic activity may be closely linked to the endogenous opioid system [181]. Moreover, OT is synthesised in peripheral tissues such as the uterus, placenta, testis and heart [182]. It is believed that plasma OT does not cross the blood–brain barrier, and there is no link between the release of OT into the blood by the neurohypophysis and local releases of OT in the central nervous system. Due to its versatile involvement in mood, stress, pain and other central nervous system effects, strong evidence exists that OT and other drugs acting through the OTR could act as multifunctional analgesics [181].

The OT signalling pathway initiates a subsequent response by the binding of OT to its receptor. The OTR belongs to the type A G-protein coupled receptor (GPCR) family and contains seven transmembrane alpha helices consisting of 389 amino acid residues. OTRs can be coupled to subunits such as Gq, Gi1, Gi2, Gi3, GoA, and GoB, causing an increase in cytosolic calcium concentration (coupling to the Gq subunit) or inhibition of adenylate cyclase activity (coupling with the Gi subunit) [183]. The OTR gene is located on human chromosome 3p25 and is about 19 kb in length. The high-affinity receptor state requires both Mg^2+^ and cholesterol, which probably function as allosteric modulators. The function and physiological regulation of the OT system is strongly steroid dependent [182].

Involvement of OT in pregnancy and child delivery is commonly known. OT helps with the cervical dilatation process and causes contractions of smooth muscle cells during the second and third stages of labour. OT is one of the most potent uterotonic agents and is clinically used to induce labour. With the onset of labour, uterine sensitivity to OT increases. This is associated with both an upregulation of OTR mRNA and a strong increase in the density of myometrial OTRs, reaching a peak during early labour [184]. Moreover, OT plays a vital role in milk ejection from the mammary gland by contracting smooth muscle cells. The secretion of the mammary glands is triggered by the tactile stimulation of receptors on site, which produces impulses further transmitted from the nipples to the spinal cord and then to the secretory oxytocinergic neurons in the hypothalamus [185]. Also, it is well documented that levels of circulating OT increase during sexual stimulation and arousal, and peak during orgasm in both men and women [186]. In addition, OT is known for reducing fear, possibly by inhibiting the amygdala [187], and its antidepressant-like effects, though it is believed that the effect may be mediated by modulation of a different target, perhaps the vasopressin V1A receptor, where OT is known to weakly bind as an agonist [188]. Moreover, OT probably has an effect in many other social interactions (i.e. generosity and empathy, trust, romantic attachment).

Interestingly, OT and PRL are able to influence each other. OT has been shown to stimulate PRL secretion when administered peripherally or directly on pituitary cells in vitro [189]. OT neurons of the paraventricular nucleus have a periodic activity that coincides with the PRL surges in rats [190], and OT directly stimulates PRL-secreting lactotrophs in rat studies through a calcium-dependent mechanism [191].

## Oxytocin and migraine

### Connection: Migraine-Trigemin ovascular System-Hypothalamus-Oxytocin

OT is a pleiotropic hypothalamic hormone/neurotransmitter since it has multiple functions ranging from mammalian behaviour and health, to neurological and immunological influences [192]. Moreover, it has an important antinociceptive role through its binding to the OTR with the effect to inhibit trigeminal neuronal excitability. However, circulating OT does not cross the blood–brain barrier, suggesting a peripheral site of action for its anti-migraine effect, likely acting on receptors within the trigeminal system. On the other side, there is a very important anatomical and pathophysiological relationship between the trigeminovascular system and the hypothalamus, of which the latter is indeed the keystone to understand the implication of OT in migraine. The role of the hypothalamus is very complex because it intervenes throughout the different phases of migraine [20]: there is an increase in hypothalamic blood flow during the presence of premonitory symptoms (nausea, vomiting, nasal congestion, and lacrimation without pain); it is involved in nociceptive processing by input from second-order neurons (trigemino-cervical complex) and output to areas of the pain neuro-axis (cortex, thalamus, amygdala, periaqueductal grey, and the spinal cord dorsal horn). Finally, disturbances of the hypothalamus lead to changes in quality of life with increased susceptibility to migraine [193].

### Oxytocin and migraine

Sensory dorsal root and trigeminal ganglia express OTRs suggesting a role in pain modulation, more specifically, an antinociceptive effect by reducing inflammation-induced firing of dorsal horn neurons and, especially, trigeminal neurons [194]. These prompted researchers to experiment with its use as a migraine drug, obviating the problem of passing through the blood–brain barrier, with intranasal administration [194, 195]. Wang et al. [196] demonstrated that repeated intranasal OT eliminates central sensitization by regulating synaptic plasticity via OTR in CM, through the downregulation of AC1/PKA/pCREB signalling pathway [197], which is activated in a CM model.

OT activates the OTR resulting in intracellular mobilisation of Ca^2+^, thereby inhibiting nociception by different actions: GABAergic signalling, inhibition of transient potassium current, desensitisation of spinal TRPV1 channels, and disruption of the NMDA-evoked coordinated neuronal network activity [198–200]. Assessing the anatomical side, the anti-migraine action follows two different pathways: projection from the hypothalamic paraventricular nucleus to the nucleus caudalis and trigeminal nociceptive pathways via activation of the OTRs.

Studies on menstrual migraine have shown that estrogen regulates OT release and the expression of the OTR [13, 23]. During menstruation there is a reduction of estrogen and OT, as well as a reduction of magnesium and cholesterol, which positively modulate the affinity of OT for OTRs. Consequences are a decrease of OT levels, reduced affinity of OT for its receptor and a decreased expression of the trigeminal OTR. All of these variations seem to determine activation of meningeal trigeminal nociceptors and increase the risk of menstrually related migraine attacks, suggesting trigeminal OTR as a therapeutic target for menstrually related migraine [14].

Krause et al. [13] demonstrated co-expression of estrogen receptors with CGRP, CGRP receptor, OT and/or OTR in migraine-related areas, namely the dorsal horn of the spinal cord, medullary dorsal horn, pontine nuclei and cerebral cortex. They also underlined the role of estrogen, which regulates the balance of pro-migraine factors, such as CGRP, and anti-migraine factors, such as OT, within the trigeminal ganglion. Moreover, OT is increased during pregnancy, coinciding with a general decrease of headaches and migraine during pregnancy [46, 201]. More specifically, this reduction in headache occurs in patients with migraine without aura, in which case there is an increase in the first trimester and a decrease in the following two trimesters, but new onset aura may appear at that time [202]. In contrast, MA patients often do not experience improvement during pregnancy and new attacks of aura without headache can occur.

### Preclinical studies

Tzabazis et al. [194, 195] investigated trigeminal ganglia applying an immunohistochemical approach in order to study the co-localization of OTR with CGRP in rats. The expression of OTRs is enhanced by painful inflammation and noxious stimulation of the face. Moreover, it was shown that trigeminal ganglion neurons possess both OTRs and CGRP and it was demonstrated that the application of OT to trigeminal ganglion neurons in vitro inhibits the firing of those neurons and the release of CGRP, with a reduction of trigeminal nerve-associated pain responses in vivo. Moreover, intranasal or intracerebroventricular OT produces a dose-dependent analgesic effect in rodent trigeminal/head pain models that is absent with intravenous application [195]. Recently, it was shown that spinal OT reduces trigeminocervical complex (TCC) neuronal firing evoked by meningeal electrical stimulation in anaesthetised rats, suggesting OTR as a possible target at the TCC level [200]. Another study [203] demonstrated that OTRs are expressed in the rat trigeminovascular system, but no OTRs in the cranial arteries were detected ex vivo [204], suggesting that the vascular effects of OT are not mediated by activation of the OTR, but through vasopressin V1A receptors instead, while OTRs are present on neuronal structures such as the trigeminal ganglion and trigeminal nucleus caudalis.

## Effect of sex steroids on oxytocin

The role of sex steroids on oxytocinergic activity has been studied extensively. Testosterone has been shown to decrease hypothalamic-pituitary-axis (HPA) activity and subsequently OT, while estrogen enhances the OTR binding in various regions of the brain in mice. More particularly, it was found that, although estrogen increases OT action via the estrogen receptor α (ERα), it also decreases its activity in an ERβ-dependent manner, having a double role. Regarding testosterone, its metabolites dihydrotestosterone and 5α-androstan-3β have been found to inhibit HPA activity via the ERβ [205]. Other studies are in agreement with these findings, so keeping in mind that steroids dramatically affect OT in most mammals, it seems likely that they have similar influences in humans as well [206–208].

This regulation of pain by OT could also be involved in menstrual migraine [13]. Decreased levels of estrogen seem to increase susceptibility for menstrual migraine attacks, and estrogen treatment in women could delay the attack until the hormone levels drop again [209]. Therefore, treatment with oral contraceptives or hormone replacement therapy that stabilises levels of estrogen may have a preventive action in migraine [13]. Pain in women with menstrual migraine, especially in those with migraine aura, usually happens at days − 2 to + 3 of the menstrual cycle, coinciding with the days of the drop of estrogens and OT [23]. Consequently, the estrogen withdrawal theory of menstrual migraine suggests that estrogen regulates the balance of pro-migraine factors, such as CGRP, and anti-migraine factors, such as OT, within the trigeminal ganglion. Regarding the role of progesterone in migraine, the results remain unclear, although it seems likely that the drop of progesterone is not related to menstrual migraine, as administration of progesterone did not protect against menstrual attacks [13].

In conclusion, the function of OT varies across women and men, proposing that the sex hormones play a role in its regulation. Keeping in mind that migraine also varies across the sexes, it can be suggested that sex hormones, OT and migraine are strongly related. Although further research is needed, such targets may be promising directions for future research in order to differentiate migraine pathophysiology and therapy in women and men [210, 211].

## Intranasal oxytocin

Intranasal delivery of OT is considered to be an attractive administration route, allowing non-invasive and rapid brain delivery of this nonapeptide by using the nasal-cerebral pathway. While subcutaneous or intravenous administration of OT is associated with a short half-life due to rapid metabolism and elimination, intranasally administered OT reaches the nervous system in larger amounts with minimal systemic exposure [212–214]. Furthermore, its targeted delivery minimises the chance of off-target (side-)effects [215]. As OT is a large hydrophilic molecule, the blood–brain barrier might hinder adequate delivery to the central nervous system of peripherally administered OT [216], despite the presence of possible transport mechanisms on endothelial cells of brain capillaries detected in mice, namely the so-called vascular receptor for advanced glycation end-products (RAGE), in both sexes [217]. Although recent studies have shown that intranasally administered OT may reach the brain in relevant amounts to exert its biological and behavioural effects [218], its site of action in migraine might not be within the central nervous system. Furthermore, while CGRP (receptor)-targeted monoclonal antibodies, for example, have a half-life of approximately one month [219], intranasal delivery allows flexibility in (daily) dosing. In general, the presence of an inflammatory environment has been hypothesised to be an essential determinant and driver of the expression of OTRs and the efficacy of intranasal OT [194, 195]. In addition, in rat studies, estrogen has been demonstrated to upregulate mRNA expression of the OTR [220]. These findings might support the hypothesis of heterogeneous responses to intranasal OT on migraine during the menstrual cycle in women and potential differences of the efficacy of intranasal OT between sexes. Further, the addition of magnesium ions to intranasal formulations appears to lead to a more robust decrease in the excitability of trigeminal ganglion neurons and to craniofacial analgesia [221].

The application of intranasal OT has already been studied in several disorders. It has been hypothesised to be a potentially safe and promising treatment option for psychiatric disorders, including autism spectrum disorder in both youngsters and adults [222–225] and in schizophrenia [226]. The therapeutic aim of intranasal OT in these disorders was to enhance social functioning, emotional recognition, and neurocognition, among others. Furthermore, intranasal OT has been demonstrated to exert its effects on the modulation and anticipation of (chronic) pain disorders [212, 227, 228]. In rats, intranasal OT reached the trigeminal ganglion and trigeminal nerves, cerebrospinal fluid, and brain regions such as the thalamus and hypothalamus [212].

The association between migraine and OT is evident when considering the association between the course of migraine and female hormonal milestones. Indeed, improvements in migraine, especially without aura, have been reported during pregnancy, lactation, and menopause [4] – events that are also associated with OT levels [24]. In addition, OTRs are expressed in trigeminal ganglion neurons, and their activation blocks the release of CGRP [194]. An older report described the pain relieving effects of OT infusion in a female presenting with MA and premature contractions of the uterus (active labour) and in a male ten-year-old presenting with acute migraine [229]. Notably, the time to pain relief was comparable to that of other vasoactive compounds—suggesting that direct cranial vasoconstriction might be the primary route of action of OT in migraine.

A pilot double-blind, placebo-controlled, single-dose study in low-frequency migraine patients showed no significant reductions of pain intensity at two hours after intranasal OT administration. Still, positive effects on other acute migraine-related symptoms (photophobia, phonophobia, and nausea) were observed [195]. A subsequent pilot study in CM patients showed a significant reduction in pain after four hours, with a lower analgesic efficacy in patients who had taken NSAIDs within 24 h [195]. The latter post-hoc findings further highlight the importance of the presence of inflammatory mediators on the efficacy of intranasal OT. Further, an open-label study including chronic and high-frequency episodic migraine patients who received intranasal OT during a period of 28 days showed a substantial decline in total headache days, with a more profound positive effect on the frequency (with a reduction of 8.2 headache days) than on the severity of headaches [195]. Another multisite double-blind, placebo-controlled study was performed in mostly female subjects suffering from high-frequency episodic or CM patients from Chile, South America, Australia, and New Zealand. These migraine patients received “as needed” chronic dosing of intranasal OT or placebo. Although a strong reduction in headache frequency was observed, an unusual high placebo response—possibly related to geographical and (socio-)cultural aspects—was observed at the Chilean site. Therefore, the latter study did not meet its primary endpoint, i.e. a reduction of migraine headache days [195].

Limitations of intranasal OT include the limited volume that can be administered, irritation and allergic reactions due to preservatives, and interference due to congestion [212], but intranasal OT shows very limited side-effects and an acceptable safety profile. These promising (pilot) results and the absence of any (serious) adverse events have made nasal OT spray an interesting prophylactic treatment option in migraine, probably also in children. Results of a phase II study of TNX-1900 (intranasal potentiated OT) for the preventive treatment of migraine in CM patients are currently awaited [230].

## Oxytocin in males

Over the last 40 years, efforts to unravel the role of ΟΤ in the male sex have increasingly intensified [231, 232]. To date, research data obtained through clinical trials highlight an important role both in the physiology of the male reproductive system, and in the behaviour of the male towards his partner and children, after childbirth [233]. With regard to its endocrine role, ΟΤ is released through the neurohypophysis into the systemic circulation during sexual intercourse and ejaculation, facilitating the secretion of sperm by inducing muscle contractions in the male urogenital system. The existence of a large amount of OT in cells of the male reproductive system (in the testicles, epididymis and prostate), raised the suspicion that it is synthesised in these organs; as a result, after numerous studies in experimental models (rats and pigs), both its paracrine role and its action at the local level were highlighted [233, 234]. Furthermore, ΟΤ has been shown to regulate androgen levels in male reproductive tissues by stimulating the conversion of testosterone to dihydrotestosterone (DHT) by 5α-reductase [235].

Since the 1980s clinical trials have been carried out in both experimental models and humans regarding the role of OT in the behaviour of men, who are the focus of most studies as they have lower levels of OT compared to women. Of great interest is the fact that ΟΤ has been shown to facilitate neurotransmission and, by extension, communication in brain areas related to emotion recognition, such as the amygdala, frontal cortex, hypothalamus and ventral area [174]. In fact, administration of OT in a double-blind study in elderly men showed an improvement in emotion recognition compared to those who received a placebo [236]. It was supposed until recently that ΟΤ increases in men after their partner gives birth, supported by a systematic review, which showed an increase in OT after childbirth [237]. However, a recent study that enrolled fathers overturned the literature of previous years, showing that OT levels are not affected by childbirth; in addition, no explanation was found for the change in behaviour of fathers towards newborns [238].

In addition to the ΟΤ action in male physiology, it is also worth highlighting its pathophysiological role in response to stress. In particular, the secretion of OT was found to occur in response to intense stressful stimuli. In a study in male participants, it was found that their exposure to industrial noise caused a significant increase in ΟΤ levels [239] and emotional stress has been confirmed as a strong stimulus for OT release in forced swimming and immobilisation rat models, in which an increase in plasma OT levels was measured [240]. Other studies have identified pain as an additional stimulus for ΟΤ release and show that corticotropin releasing hormone (CRH) mediates OT release in various stress models. Significant increases in ΟΤ levels have also been measured during insulin-induced hypoglycemia and during bowel manipulation while abdominal surgery was being performed, confirming that painful stress also activates the OT release system in humans [240].

In recent years, based on all the knowledge that has emerged about the role of ΟΤ in the male reproductive system, efforts are being made to develop pharmaceutical interventions targeting OTRs. The first studies were conducted in male sheep in vivo, in which ΟΤ administered under general anaesthesia was found to cause an increase in both fluid quantity and sperm count [241]. Today, there is an attempt to create OTR agonists for the treatment of pathologies related to male reproductive function, sperm improvement and for the treatment of prostate disorders [242]. In particular, the discovery of the presence of OTRs in the male hyperplastic prostate led to the conduct of studies with the local use of OTR agonists with the aim of both symptomatic treatment and halting the progression of the disease [235]. Moreover, OT expression has been found to decrease with the progression of prostate cancer, potentially becoming a future biomarker for invasive disease [243].

### Therapeutic use of oxytocin in males

The analgesic effect of OT has been tested in humans many times. In 1995, a study including 48 healthy male volunteers found that inhaling OT spray reduced the pain threshold by 56.5% [244]. A more recent study investigated the analgesic effect of OT on pain in both men and women. A reduction in pain perception after intranasal OT application was observed in men but not in women. One reason may be that women are more sensitive to psychological or social stimuli of stress or pain than men as well as the fact that in women endogenous levels of OT vary during the menstrual cycle [245]. Additionally, another study investigated the effect of OT on CM in male mice. It was found that repeated administration of intranasal OT significantly reduced pain through the OTR, while the expression of CGRP and c-Fos was also found to be reduced, thus indicating the role of OT in preventing central sensitization. Therefore, intranasal OT is a potential treatment for the prevention of CM [197], and could possibly be effective in both men and women.

## Interaction of prolactin and oxytocin

The relationship between OT and PRL described above is based on the interaction in various life processes. Rat models revealed that OTRs are present on lactotrophs and in response to endo- and exogenous OT, they activate lactotrophs and increase PRL secretion. OT can, therefore, be regarded as prolactin-releasing factor. OT may reach the lactotrophs through the long portal vessels, the short portal vessels and from the peripheral circulation (OT release from the posterior pituitary) [246] via a Ca^2+^-dependent mechanism [191]. During breastfeeding, in response to sucking, the level of OT in the peripheral blood increases first, followed by the secretion of PRL. This suggests involvement of other regulating mechanisms and different thresholds of activation [247]. It seems that OT acts peripherally to stimulate PRL secretion, while PRL acts on OT through central mechanisms [248]. Administration of an OT antagonist with a fixed dose causes no increase in PRL levels in response to sucking, indicating an effective blockade of the secretion [79, 249, 250]. An inverse relationship is observed during lactation, when PRL stimulates the release of OT. This may be due to the existence of a positive feedback loop between the two hormones and mutual stimulation of secretion [251]. Disrupting the rhythmic release of PRL through an OT antagonist will also inhibit the rhythmic release of dopamine [248, 252]. Moreover, it seems that OT and dopamine may affect the circadian rhythms of PRL secretion in animal models and possibly the estrogen cycle [253].

In humans, both hormones are in part mediated through distinct mechanisms. Positive OT and PRL correlation was observed during breastfeeding. Notwithstanding, during this episode, OT is released in a pulsatile pattern and within minutes, while the increase in PRL secretion is slower and the effect lasts longer. Milk production and ejection is stimulated by OT via oxytocin-containing nerves from paraventricular nucleus to PRL-producing cells in the anterior pituitary [254]. OT and PRL are also both involved in pair-bonding and infant care [255].

## Discussion

### OT and PRL in migraine pathophysiology

Over the last 50 years, pain research has provided considerable support for the assertion that the hypothalamic peptide OT and pituitary peptide PRL affect pain modulation in animals and humans. It seems that the analgesic effect of OT and female-specific hyperalgesia of PRL play a key role in this process [14, 31, 78, 81]. Research on PRL revealed that this endocrine hormone is linked with migraine as well as many other types of pain. On the other hand, low levels of OT in humans may be a component of many chronic pain conditions and have been associated with increased sensitivity to pain [16, 17, 81, 181, 256].

**Fig. 1:**
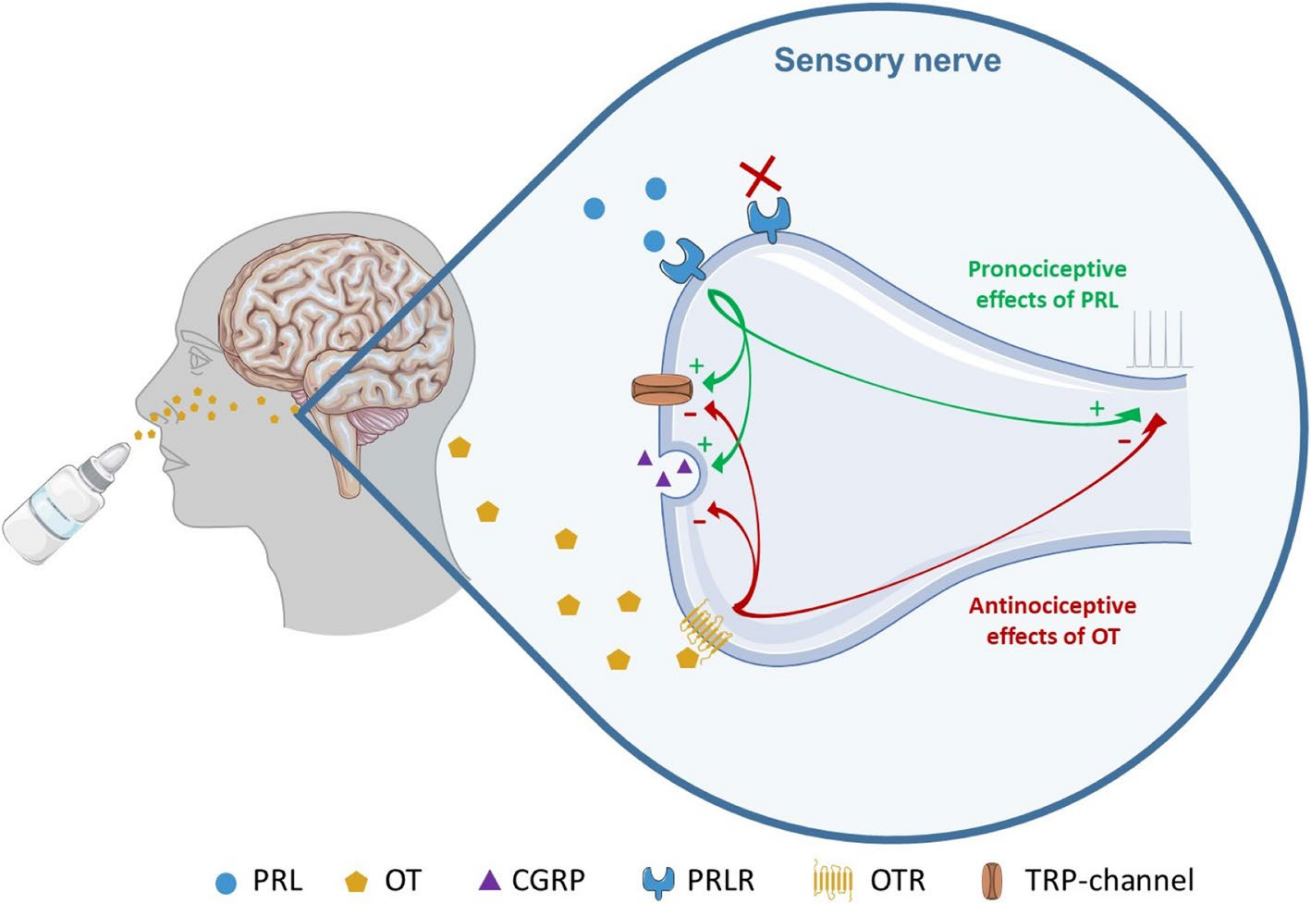
Effects of OT and PRL on sensory nerves in the trigeminal system. Intranasal OT can reach the trigeminal system and activate the OTR, resulting in inhibition of CGRP release [195], desensitisation of TRPV1 channels [199] and inhibition of neuronal firing [200], leading to antinociceptive effects. PRL activates the PRLR, resulting in increased neuronal excitability [14, 99], sensitization of TRPV1, TRPM8 and TRPA1 channels [33, 79, 102, 105], and release of CGRP [15, 82], suggesting that treatment targeting PRL should aim to decrease its pronociceptive effects, e.g. using PRLR antagonists or decreasing PRL levels

Clinical and preclinical studies have postulated a role for PRL and PRLR in headache disorders, particularly migraine. In the nervous system, PRL acts on various neuronal circuits, including trigeminal sensory neurons, through binding to PRLRs, where it increases neuronal excitability [78]. The effects of OT can be explained by binding to the OTRs, as well as passive diffusion into various brain structures. Moreover, its activity may also be closely related to the endogenous opioid system [257]. Identically, PRL and OT are involved in nociception, acting at peripheral and central levels, with the distinction that PRL is pronociceptive, and OT is antinociceptive [78, 79] (Fig. 1). In this respect, OTR activation on sensory neurons inhibits neuronal firing [200] and leads to desensitisation of TRPV1 channels [199], while PRLR activation on sensory neurons increases neuronal excitability [14, 99] and sensitises TRPV1, TRPM8 and TRPA1 channels [33, 79, 102, 105].

PRL can access the nervous system, probably through two mechanisms: mediated by receptors or through regions that lack a blood–brain barrier, while OT is only able to pass into the central nervous system where the blood brain barrier is not present [16, 78, 218, 256, 258]. Another important issue is that PRL may be regulated by systemic processes such as hormones (estrogens) or inflammation [78–80] and OT levels can be regulated by parietal or sexual behaviour and modulate psychological functions [186, 259]. Considering the location of the receptors on which these hormones exert an effect, PRL is mediated by PRLR in pain-associated structures, and, although more specific data for the OTR are lacking, researchers documented its highest concentration in the lower medulla where the trigeminal nucleus caudalis is located [25]. It is confirmed in animal studies that in trigeminal neurons CGRP is also co-localized with both OTR and PRLR [15, 17], where activation of the OTR results in inhibition of CGRP release [195], while PRLR activation stimulates the release of CGRP [15, 82]. The inflammatory molecule PACAP-38, another significant inflammatory peptide, also co-localises with PRL, and during the pain this peptide increases PRL release [88]. In the hypothalamus, PACAP partially colocalizes with OT as well [260].

### PRL as a target for the treatment of migraine

Studies on PRL levels almost comprehensively indicate that this hormone is found at higher levels in migraine patients compared to healthy controls [31, 80]. Furthermore, it has been suggested that high PRL levels play a role in the chronification and progression of migraine. Clinical studies in patients with hyperprolactinemia demonstrated that headache is decreased, in some cases diminished via downregulation of PRL [25, 111, 261]. It has been demonstrated that some migraine medications have an impact on PRL levels [22, 152, 153]. Acetaminophen, triptans and propranolol decrease PRL levels, while treatment with valproate increases PRL levels. Previous studies showed that drugs such as bromocriptine and methysergide, which affect PRL levels, are effective in the prevention of migraine, presumably by interacting with dopamine and 5-HT receptors. However, their use in migraine is discouraged due to the risk of causing serious side effects like pulmonary fibrosis.

In terms of the inflammatory side of migraine, PRLR is known to be expressed on CGRP-immunopositive sensory fibres and promotes the release of CGRP. Additionally, the CGRP receptor is expressed on immune cells, including dural mast cells, macrophages and T-cells, and CGRP may interact with these cell types to stimulate the release of PRL [15]. This reciprocal link is also associated with a female-specific migraine-like behavioural response, which decreases in the presence of CGRP_8-37_, a CGRP receptor antagonist [15–17, 82, 262]. PACAP-38, another inflammatory agent that may have an accent role in migraine pathogenesis, has been shown to cause increased PRL release in MO patients [88].

Besides the direct link between PRL and (migraine) pain, the relationship between inflammation and PRL raises the question whether blocking PRLR can be used in migraine pain—not only as a preventive but also as a treatment that targets pathophysiological mechanisms of migraine. PRLR antagonists are suggested therapeutics for certain malignancies but there is no data in the literature related to pain. The PRL system could be targeted in various manners, such as by targeting phosphorylation or dimerization of the receptor [263]. Nevertheless, the PRLR has been shown to have single nucleotide polymorphisms, some of which may influence how the receptor functions and reacts to receptor blocking [264]. Furthermore, monoclonal antibodies against PRL could possibly be used to counteract its effects. However, considering the many different actions of PRL and its diverse physiological role, preferably only the nociceptive effects of PRL should be counteracted, to avoid adverse effects. Following this rationale, treatments targeting PRL nociception should specifically target the PRLR in certain migraine pain-related structures, such as the cranial trigeminal system. This could be done using locally applied treatment, as is done for OT using an intranasal spray, or using treatment that specifically targets one area or one type of tissue or cell, for instance specifically targeting nociceptive fibres or mast cells. The field of tissue-specific drug delivery is still under development, but gives great hope for treatment in the future [265].

Preclinical studies have shown that PRL contributes to the pathogenesis of migraine involving sex-specific mechanisms [16]. In animal studies, PRL administered to the dura mater elicited migraine-like behaviour only in females, and PRLR was more abundant in the female sex in trigeminal neurons [78, 79]. We can also claim that PRL-induced sensitization of TRPV1, TRPM8 and TRPA1 channels in dorsal root ganglion neurons [14, 15, 78, 79] and trigeminal ganglion neurons [33] is a sex-dependent mechanism, given that the induced increased calcium influx is only observed in females and not in male neurons [88]. In addition, behavioural animal studies have shown that PRL can induce nociception in female rats but not in ovariectomized rats [33], suggesting that PRL-induced nociception effects are estrogen-dependent [33]. Lowering PRL levels is likely to cause sexual side effects in males. In the light of this information, it seems that blocking PRLR should be considered as a preliminary objective in females rather than males. Moreover, considering that the PRLR is also present in pain-related centres in males but is detected in much higher concentrations in females, blocking some of the receptors involved in nociception rather than all receptors might be a preferred option in terms of reducing side effects.

Recently, it was shown that PRL could be targeted more upstream as well, using kappa opioid receptor (KOR) antagonists. Stress-activated hypothalamic KORs were shown to increase PRL levels resulting in trigeminal sensitization, with effects in both male and female mice, suggesting that these KOR antagonists could potentially be used for migraine prophylaxis in both sexes, while decreasing PRL levels using the dopamine receptor agonist, cabergoline, seemed to be effective for reducing allodynia in only females, without any effect on males [125].

### OT as a target for the treatment of migraine

OTR upregulation in the trigeminal ganglion after noxious stimulation and a blockade of capsaicin-induced CGRP release from trigeminal dural afferents (nociceptive primary afferent neurons) after exogenous OT was observed [194]. Also, in a rat model of traumatic brain injury, intranasal administration of OT into the trigeminal ganglia attenuated allodynic responses, while no effect was seen after intravenous OT injection of the same dose. In this study, OT concentration was measured in the spinal cord, trigeminal ganglia, pons and the olfactory bulb. After intranasal administration, a higher concentration of OT was observed in all these areas, however, statistical significance was reached only in the trigeminal ganglion. Those results rather contradict the activity of OT in the peripheral blood stream, and support central transmission of OT [266]. Also, in the nucleus accumbens, reward-related processing due to modulation of dopaminergic reward pathways between OT and dopamine receptor binding have been observed [267], which is important for coping behaviours. The results of above studies allowed us to hypothesise the central role of OT in migraine pathophysiology via descending hypothalamic pathways (transport via olfactory and trigeminal nerve fibres) and/or peripheral processing via circulating OT in the blood [196, 204]. The fact that the OTRs in the trigeminal ganglion are located outside the blood–brain barrier makes them available as potential targets of intranasal OT or OTR agonists to counteract migraine headaches in men and women.

OT at intranasal (or cerebroventricular) application inhibits the nociceptive activation of neurons in the trigeminal nucleus caudalis, and its added advantage is a dose-dependent and specific response [195]. OT is a linear organic polymer, which is not likely to cause addiction and has low toxicity and strong potency. As disadvantages, inadequate deposition of functional peptide to specific brain regions and short half-lives and poor availability when administered orally or parenterally, may be mentioned [212, 268]. The role of chronic OT use in stress and anxiety reduction as well as long-lasting effects on cardiovascular responses were reported. These reactions were dose-related and depend on contextual (such as developmental period or the type of stress experienced) and interindividual (e.g. sex, genotype) factors [269]. The above remains relevant when considering trigger factors for migraine headaches; however, potential positive, indirect, effects on headaches may also be connected to improvement in sexual functions [270] and social interactions, which positively affect the quality of life.

Besides OT, other OTR agonists could be used to target the OT system. There are limited reports, which provide long-lasting reduction in inflammatory pain-induced hyperalgesia symptoms after intraperitoneal administration of a non-peptide full agonist of the OTR (LIT-001) in animal models [271], or antinociceptive effect of carbetocin, an OTR agonist, in humans [272]. Further research is in progress, however, the effectiveness of the agonist may depend on selectivity and its route of administration [273]. Nevertheless, genetics [274, 275], sexual differences [276], and stress-related psychopathology [277] should be taken into account when making therapeutic decisions. Sexual dimorphism on OT and OTR expression remains significant and makes women more vulnerable to OT decline and an associated greater possibility of developing depression or menstrual pain [24]. Increased endogenous OT level is recorded between the attacks due to generalised enhancement of affective pain and neurogenic inflammation. This increase is probably a response to migraine stress, however, compensatory mechanisms to reduce affective stress are also suggested [278].

### Interaction between OT and PRL

The interaction between OT and PRL needs to be considered as well. In this respect, OT can be regarded as a prolactin-releasing factor [246], while PRL can stimulate OT secretion through central mechanisms [248]. The fact that the levels of one of these hormones can affect secretion of the other should be taken into account when using them as a target for migraine treatment, especially considering that OT has antinociceptive effects, while PRL leads to a more pronociceptive response. Treatment should aim to increase OT levels, thereby increasing antinociceptive effects, without resulting in subsequent increased PRL levels, which could potentially counter the antinociceptive effects of OT. This interaction between OT and PRL levels stresses the need for a locally applied treatment, which is realised with the intranasal application of OT.

## Conclusions

To gain insight into the role of OT and PRL in migraine pathophysiology, we have focused on their effects on pain modulation. Both hormones are involved in nociception, operating at the peripheral and central levels; however their role is the opposite. PRL, despite its role in many physiological processes, has a pronociceptive effect (remarkably in the female sex) and may contribute to recurrence or chronification of pain symptoms. In contrast, OT is characterised by antinociceptive activity, which is particularly marked during pregnancy, feeding and/or sexual behaviour in both sexes. In connection with this discovery, an increasing role is attributed to drugs that can act on their receptors. Additionally, the fact that they co-localize with estrogen receptors, CGRP and CGRP receptors provides great hope for influencing nociceptive pathways.

The relationship between various sex hormones is complex and their interactions should be taken into account. Despite this complexity, the data shown above indicate a clear influence of PRL and OT, as well as other hormones and peptides, on the occurrence of migraine. These dependencies consist of intricate mechanisms which, thanks to the increasing number of studies, are becoming better understood. The role of the hypothalamus, a part of the migraine-related regions, as the initiator of migraine is also increasingly emphasised. While migraine is more prevalent in women, it should also be taken into account that there is insufficient data on the role of sex hormones in males, and this topic needs more research. Also, research into the correct administration of doses according to sex-specific differences, hormonal changes and comorbidities remains a major challenge for the development of future therapies.

## Abbreviations

5-HT: Serotonin
CGRP: Calcitonin gene-related peptide
CM: Chronic migraine
CSD: Cortical spreading depression
FHM: Familial hemiplegic migraine
FSH: Follicle-stimulating hormone
GABA: Ƴ-Aminobutyric acid
GH: Growth hormone
GPCR: G-protein coupled receptor
KOR: Kappa opioid receptor
LH: Luteinizing hormone
MA: Migraine with aura
MO: Migraine without aura
OT: Oxytocin
OTR: Oxytocin receptor
PACAP-38: Pituitary adenylate cyclase activating polypeptide-38
PRL: Prolactin
PRLR: Prolactin receptor
TCC: Trigeminocervical complex
TRP: Transient receptor potential cation channel
TRH: Thyrotropin releasing hormone
